# The History of Cataphoresis Corrected

**Published:** 1896-10

**Authors:** D. F. McGraw

**Affiliations:** San José, California


### Domestic Correspondence.

### THE HISTORY OF CATAPHORESIS CORRECTED.

To the Editor:

Sir,—In view of the many discoverers of cataphoresis, or, more properly speaking, electrolytic obtunding of sensitive dentine, I wish to call the attention of the dental profession to the following extract from a paper that I read before the Minnesota State Dental Society at their annual meeting in 1888; also read at the Southern Minnesota District Society at their semi-annual meeting in the fall of 1888; also was read at the Twenty-fifth Annual Meeting of the Chicago Dental Club, held in the Grand Pacific, on February 5, 6, and 7, 1889, by the secretary of that club, in view of my enforced absence. The transactions of that meeting in which the article appears were published in the February number of the *Dental Review;* subsequently they were published in book form, and each member and visiting member was furnished with a copy. The article appears on page 113 of these transactions.

Professor Weeks of the Dental Department of the University of Minnesota read a very interesting article on this subject at this same meeting, as we were working on these same lines, his attention having been called to the subject by the papers I read at this meeting in the summer of 1888. The subject was discussed at some length by Drs. Custer, Atkinson, Thompson, Harlin, Baldwin, and Taft.

The difference in my present method is to make the current constant from nine to fifteen minutes as the needs of the case may suggest. The formula is one that any dentist can compound, and any good galvanic battery of twelve cells will do the work.

### EXTRACT.

"This method that we shall present to you at this meeting to gain insensibility in the teeth is the following:

"To a twelve-per-cent. solution of cocaine add an equal amount of absolute alcohol, making a six-per-cent. solution of cocaine in alcohol. In connection with this I use the galvanic current, varying the power as the needs of each case may indicate. The method of application is as follows:

"First apply the rubber dam; wet a pledget of cotton in the solution, placing it in the cavity of the tooth, pressing the point of the positive pole onto the cotton, and the negative pole, with sponge attachment thoroughly wet, to the cheek, turning on the current. Rarely will more than four cells be necessary, if the battery is in good working order.

"An application of three minutes, with an interval of three minutes, and then another three minutes application, are sufficient in the majority of cases, although I have to occasionally make the third application, then dry the cavity thoroughly and commence excavating. My deductions as to the physiological effects are as follows: The galvanic current acts as a vehicle for conducting the medicinal agents; the cocaine anæsthetizes the odontoblastic cells and the pulp; the styptic properties of the alcohol acts upon the dentinal fibrils, they being of an albuminous nature, causing contraction and increased density and firmness.

"My reasons for drawing these conclusions are these:

"I have found that the most sensitive teeth can be obtunded; that after a certain period of rest sensitiveness returns, but never to that degree that existed before the application of the obtundent. Therefore I conclude that a change had taken place in the dentinal fibrils, which I maintain is due to the styptic properties of the alcohol and not to the electrolytic actions of the galvanic current. Another reason is, that a tooth in which the pulp is devitalized is a non-conductor of the electric current. A tooth which has been extracted was subjected to a twelve-cell current of a freshly charged battery and proved an absolute non-conductor.

"In the treatment of peridental inflammation we have to use a stronger current, for this reason. It is well known that a strong current will tetanize the vessels, causing a diminished flow of blood to the parts, thus lessening congestion. The same current longer continued will cause electrolytic decomposition. These are laws of galvanic electricity that are incontrovertible. The medicinal agents that I use in all cases of peridental inflammation and the blind abscess are as follows:

"A saturated solution of chloride of sodium, seven ounces; tincture of ergot, one ounce. In the chloride of sodium we have one of the constituents of the blood where it keeps the fibrin and albumen in solution. The tissues in an inflamed condition lack this element, which we supply artificially. In the tincture of ergot we have a drug that stimulates contraction of the blood-vessels, causing anæmia. Taken together we have here a combination which decreases the flow of blood, reducing congestion, at the same time furnishing an element which is lacking and upon the presence of which normal conditions depend.

"The treatment of blind abscess requires stronger battery power, in order that we may get the full benefit of electrolysis.

"Dr. Weeks informs me that he has had remarkable success in using this method in removing pulps. My experience in this line has been exceedingly limited, having used it in only two cases. The first was partially successful, and the second was a complete success. The failure of the first I attributed to the congested condition of the pulp. I would recommend getting the pulp in a healthy condition first, as I find that in cases where we have an inflamed condition, the full anæsthetic effects are not as easily gained as where the pulp is normal."

Very respectfully,

D. F. McGraw.

San José, California, July 25, 1896.

